# The Influence of Image Realism of Digital Endorsers on the Purchase Intention of Gift Products for the Elderly

**DOI:** 10.3390/bs13010074

**Published:** 2023-01-15

**Authors:** Xiaoyi Wang, Xingyi Qiu

**Affiliations:** 1 School of Management, Zhejiang University, Hangzhou 310012, China; 2 Neuromanagement Lab, Zhejiang University, Hangzhou 310012, China

**Keywords:** elderly product, digital endorser, advertising, perceived social value, framing effect

## Abstract

Digital endorsers are already utilized extensively in various businesses. The major objective of the current study was to find out the impact of image realism of digital endorsers on the purchase intention of gift products for the elderly. We investigated this issue through three online experiments. Study 1 (*n* = 205) found that cartoon digital endorsers (vs. realistic digital endorsers) generate higher purchase intention for the product. Study 2 (*n* = 175) showed that perceived social value plays a mediating role in the relationship between the image realism of digital endorsers and purchase intention. Study 3 (*n* = 127) demonstrated the moderating role of information framing in the relationship between the image realism of digital endorsers and purchase intention. In all, our research extends the previous literature on digital human endorsements and advertising of elderly products and provides several managerial implications for consumers and marketers.

## 1. Introduction

As the aging of the population is projected to accelerate around the world [1], more and more products for the elderly are being introduced to the market. Although younger endorsers have greater appeal [2], according to the “Match-Up” hypothesis [3], the use of age-mismatched endorsers can make advertising less persuasive and expressive [4,5]; therefore, it is often best to use an elderly endorser when the product is aimed toward the elderly [6]. However, at the same time, unfavorable stereotypes about old people in general culture can undermine positive views of the product [7]. Therefore, advertisers typically use younger elderly [8] or hire younger people to pretend to be older people for their endorsement advertising to increase the effectiveness of the ads [9]. In recent years, digital endorsers have also been adopted in the advertising of products for the elderly, for example, in famous health products such as Naobaijin (Wuxi Jiante Pharmaceutical Co., Ltd.) and Hidrocon Immune Capsules (Shenzhen Kangmei Industrial Development Co., Ltd.), and set sales records to the tune of several billion CNY [10].

The phenomenon of digital characters first emerged in the 1990s, and cartoon characters were the first digital characters to be used in advertising [11,12,13]. Around 2016, more advanced technology enabled the emergence of a group of digital characters with a more realistic human look. These high-form realism digital endorsers quickly entered the endorsement activities of well-known brands [14,15]. There are conflicting assertions regarding whether the increased realism of digital endorsers’ image would have a positive or negative effect on advertising. On the one hand, a high level of image realism improves the perceived ability of digital endorsers and hence raises consumer positive attitude [16]; On the other hand, a high form of realism is associated with a low construal level, which is not conducive to consumers’ overall perception of the face of the endorser to form a coherent and stable cognition [17]. As more digital endorsers appear in advertisements of products for elderly people, how will the degree of their image realism affect consumers’ purchasing intentions? This is the important focus of this research.

Digital endorsers’ varying levels of image realism result in various construal levels, which further drives consumers to have various understandings [18]. The degree of cognitive consensus will cause a shift in perceived social value [19]. Products for the elderly are frequently seen in gift-giving scenarios to demonstrate young people’s concern for their elders and the emotional bonds that exist between families. Perceived social value is a crucial factor for buyers in the gift-giving scenario [20]. Therefore, this research proposed that perceived social value plays a mediating role in the relationship between the image realism of digital endorsers and purchase intention. In addition, adjusting the information framing of advertising content can influence consumer perceptions [21]. Preventive information framing can reduce consumers’ intuitive judgments based on digital endorser images [22]. Hence, we introduced the variable of advertising information framing as moderators to alleviate the negative impacts of high realism in digital endorsers.

The present article is structured as follows. Section 2 reviews the related literature and puts forward the research hypothesis. Section 3 briefly introduces the methodology and design of three experiments. Section 4, Section 5 and Section 6 explain the three studies’ procedures, results, and conclusions in detail. Section 7 and Section 8 discuss and conclude the research results. 

## 2. Literature Review and Research Hypotheses

### 2.1. Endorsers of Products for the Elderly

The published research investigates the effectiveness of human endorsers from two major theoretical perspectives. One is the source-credibility theory [23]; endorsers with higher credibility are more widely recognized and more popular, and have a greater impact on changes in consumer attitudes and purchase intentions [24,25,26]. The most common three-dimensional source-credibility models include expertise, trustworthiness, and attractiveness [9,27,28]. On the contrary, the negative attributes of the endorsers might also adversely affect consumers’ attitudes toward the product [29,30,31]. The other is the “Match-Up” hypothesis [3]. Endorser–product congruence enhances the transfer of perceived attributes from endorser to product [32]. Congruence has a considerable positive association with brand attachment, brand commitment, and brand loyalty [33,34].

Although younger endorsers are more appealing [2], according to the “Match-Up” hypothesis [3], advertising may be less compelling and expressive when using age-inappropriate endorsers [4,5]; therefore, it is often best to use an elderly endorser when the product is aimed toward the elderly [6]. The classification of the elderly group has been more detailed in clinical medicine research and consumer behavior research [35,36,37]. Each type of the elderly has unique characteristics and behaviors [8,38]. At present, the elderly appearing in advertisements are mainly the young elderly. The elderly over 70 years old and the super elderly have been ignored by advertisers. While advertisers try to show the advantages of elderly endorsers, there are still many opposing viewpoints regarding the representation of the elderly in advertising. Elderly endorsers are typically portrayed as rather prestigious characters in non-elderly product advertisements [39]. For instance, knowledgeable tutors and affluent retirees [40]. At the same time, elderly endorsers are frequently connected with negative stereotypes, such as physical aging, cognitive decline, depression, and a lack of beauty [2,41], and they are frequently portrayed as in need of assistance [42]. As a result, elderly endorsers are rarely pictured in traditional ways in most advertisements for products geared toward older consumers. Instead, advertisers utilize the ads’ visual and textual components to downplay ageism, hoping the elderly in the ad are seen as “the eternally youthful seniors” [43]. Companies and consumers have conflicting attitudes toward elderly endorsers because they need to maintain the traits of older people to show congruence between the endorsers and the product, but do not want negative associations about older people to harm sales.

A wide variety of products for the elderly have emerged as a result of the aging of the population. Previously, studies focused on the back end of the consumption chain in terms of design and functional benefits [44,45,46], whereas few studies focused on the front end of the consumption chain in terms of how to stimulate the purchase intention of products for older people [8], e.g., research on marketing and advertising [47]. The need for elderly product endorsers is gradually rising; however, there is a shortage of elderly endorsers. On the one hand, elderly endorsers are hard to come by, and the majority of well-known older celebrities are wary about endorsing elderly products due to the potential for unfavorable connections [48]. In the fashion industry, only 1 out of every 200 models is over the age of 50 [49]. On the other hand, due to health issues for the elderly, advertising design with real elderly people as endorsers takes increased risk throughout the filming process, making it harder to guarantee the quality of the advertisements [50]. Therefore, we propose introducing a new type of endorser—the digital endorser—to be involved in the promotion of products for the elderly.

### 2.2. The Form Realism of Digital Endorsers

**Figure 1:**
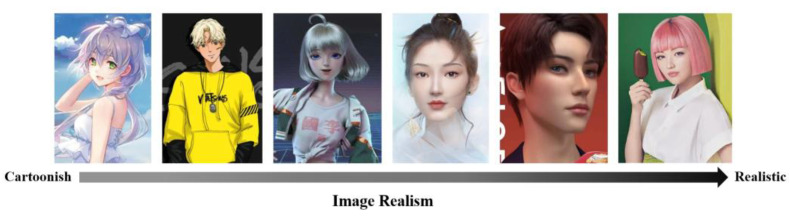
Example of digital endorsers’ image.

The digital human is an integrated product with multiple human characteristics that is created using computer tools such as computer graphics, deep learning, and speech synthesis [51,52]. A digital endorser is an identity for a digital human when they are involved in brand and product marketing activities [53]. Compared to human endorsers, digital endorsements have many benefits: Firstly, they are more flexible, adaptable to different creative contexts, and not limited by space or time [54]. They interact with customers more frequently, resulting in greater immersion [55,56]. Secondly, they keep away from misbehavior and negative news [57]. Thirdly, companies and products using digital endorsements are regarded as being more innovative and technological [58,59]. The digital human can exist in nearly any form in different parts of the world, build their own digital lives and participate in value co-production [60,61,62]. Research has been conducted on the image of digital endorsers, their interactive behavior, and their compatibility with the product [63,64,65,66]. According to the degree of realism in the image, digital humans can be divided into two categories: the more abstract Cartoonish digital endorser and the more human-like Realistic digital endorser [53,67,68,69], as shown in Figure 1.

The main difference between the two types of digital endorsers is the degree of realism in their appearance [53]. The cartoonish digital endorser’s image is more abstract. When confronted with new, unfamiliar objects, people frequently engage in a high construal level, generate quick impressions based on scant information, and pay greater attention to the essential characteristics of objects [18]. A high construal level enables people to broaden their mental perspectives, extend in-group borders, and comprehend things in an abstract, holistic manner [70]. The more tangible and real the image of the realistic digital endorser, the less consumers regard it as an abstract figure with a steady personality [71], and the more they focus on the realistic digital endorser’s genuine qualities and various nuances. This causes people to narrow their mental horizons, concentrate more on urgent demands, become more easily influenced by others, and become less likely to have a solid and cohesive view of digital endorsers [72], leading to more varied perceptions among customers [73].

Consumers’ primary cognitive mechanism for endorsers is facial recognition [17]. Facial recognition in advertising is a transient social assessment [74], also known as thin-slice judgment [75], in which people utilize only a tiny quantity of information to create a rapid judgment about the face they are recognizing [76]. This is an instinctive emotional reaction that is devoid of logic and reasoning [77]. While the cartoon digital endorser’s construal level is higher, consumers seek a “bigger picture” when generating an impression of it; that is, a holistic perceptual processing of the face, rather than simply depending on a specific characteristic [17,78]. Images with a high construal level also help reduce prejudice and stereotypes [79]. Therefore, we propose the following:

 **Hypothesis 1.** *Cartoonish digital endorsers generate higher purchase intention than realistic digital endorsers in the advertising of products for the elderly.*

### 2.3. The Mediating Role of Perceived Social Values

Brands constantly consider what values consumers desire and how to obtain them to receive a competitive advantage, since consumers are “value-driven” [80]. While satisfaction and perceived value both exist at different phases of the purchasing process [81], it is widely agreed that satisfaction is evaluated after the sale and after use [82], but perceived value arises before the sale, and buyers can generate perceived value during the advertising stage [83]. Perceived value has continuously grown from unidimensional to multidimensional [84,85], with economic, functional, emotional, and social values being the four most commonly employed [85,86,87].

One of the most crucial considerations for buyers when purchasing gifts for the elderly is perceived social value [88]. Personalization can offer a unique symbol of meaning [89], but it is not without risk. The more specific the gift, the more likely the gift recipient will match the gift to the target identity [90]. When the gift is incongruent with the recipient’s identity, anxiety and bewilderment can escalate [91]. Young people are typically driven more by social emotions [92] than by utilitarianism when they give gifts to their seniors [93]. As a result, the meaning of the gift must not be misconstrued or ambiguous, as this will impede the giver’s expression of social identity [94]. Consumers might gain social recognition or enhance their social self-concept when they buy and use products or services, which is known as perceived social value [85]. According to realistic conflict theory [95], when attitudes and ideas differ between groups, it is difficult to subjectively regard oneself as belonging to others and therefore build a sense of identity. The higher the construal level of the target object, the more likely people are to identify with others, improving social identity and increasing perceived social value [19]. In conclusion, abstract cartoon digital endorsers elicit higher perceived social value, which in turn boosts consumers’ positive attitudes toward the advertised product and their faith in the concrete results that the product and service would bring [96].

 **Hypothesis 2.** *Perceived social value mediates the impact of the type of digital endorser on purchase intention.*

### 2.4. The Moderating Effect of Advertising Information Framing

Realistic digital endorsers can have a wide range of uses in more scenarios [97]; therefore, it is important to figure out how to mitigate the negative impact of high image realism. Through different expressions or wordings of the same information, the framing effect enables the information’s receiver to perceive, judge, and arrive at alternative conclusions about the object being described [98,99]. Different types of information framing have a strong impact on advertising [21]. Among them, positive framing (PF) highlights the benefits of engaging in a particular behavior, whereas negative framing (NF) stresses the loss of not engaging in a certain behavior [100,101,102]. The dimension of attention and understanding of information before people make judgments is influenced by information framing [103]. When faced with risks, people typically overlook the factors that have relatively modest changes in outcomes and only take into account those factors that have significant disparities in outcomes. 

Many existing studies demonstrate interactions between information framing and construal level, with positive information framing being more compelling among consumers with high construal levels [104]. In promotional framing, the effect of the construal level on risk decision making is significant, but not in preventive framing [105]. Therefore, when product advertisements are presented in a promotional framing, consumers will rely more on intuitive judgments and ignore objective conditions [106], thus producing more positive results for information with a high construal level [107]. When advertising contents are presented in a preventative framing, the endorser matches better with the description of the product message, enhancing consumer connection and attention to the ad [108,109]. Consumers can initiate a logical, rational way of thinking by moving their focus to the probability of a positive outcome, carefully assessing and evaluating information about products and endorsers in advertising [22], and thinking about both the advantages and negatives from multiple viewpoints. Thus, by adjusting the information framing of the advertisement, it is feasible to alleviate the negative impacts of the realistic digital endorsers and enhance the amount of customer concern about its merits [110]. As a result, we propose the following hypothesis:

 **Hypothesis 3.** *The advertising information framing moderates the relationship between the type of digital endorsers and purchase intention: in the promotional information framing, cartoon digital endorsers lead to higher purchase intention; in the preventive framing, there is no significant difference in purchase intention between the two types of digital endorsers.*

## 3. Materials and Methods

The data of this research were collected by online experiment. The online experiment method is one of the most common research methods used by scholars in the field of consumer behavior [111,112,113]. Besides lower cost, another advantage of online experiment is that they are more scalable in terms of number of participants [114]. It is even possible to run short experiments with many participants at the same time.

### 3.1. Design of Stimulus Materials

We chose photos of ordinary elderly people as the original material, based on which we processed them using computer image generation software to obtain pictures of realistic digital humans and cartoonish digital humans. After adjusting elements such as clothing and picture size, the experimental materials of the three groups of endorsers were obtained. The main difference in the experimental material was the head and face performance of the endorsers; according to the previous concept that face information is a rich source of thin-slice judgments [115,116], the review and estimate of the endorser’s face was sufficient to influence consumer attitudes [117], and therefore no significant changes were made to the part of the endorsers below the head. Pictures of the specific stimulus material will be presented in subsequent studies.

### 3.2. Overview of the Studies

According to the paradigm of consumer behavior research [118,119,120], we use three studies to investigate and analyze the core problem of this research. In Study 1, we examined the main effect of how digital endorsers with a low degree of image realism can increase consumers’ purchase intention. In Study 2, we used mediation analysis to confirm that the degree of digital endorsers’ image realism affects purchase intention through perceived social value. In Study 3, we tested whether the information framing can moderate the impact of the degree of digital endorsers’ image realism on the purchase intention through moderated mediation analysis. We recruited participants on different online platforms in three studies and adopted our measures from existing studies. The statistical analysis software SPSS 25.0 was used to analyze the results.

## 4. Study 1

In Study 1, we expected to verify two fundamental phenomena: firstly, the digital endorsers meet the requirements of age selection of endorsers for the product for the elderly (the commercial practice); secondly, cartoonish digital endorsers (vs. realistic digital endorsers) lead to higher purchase intentions (H1).

### 4.1. Participants and Procedures

**Figure 2:**
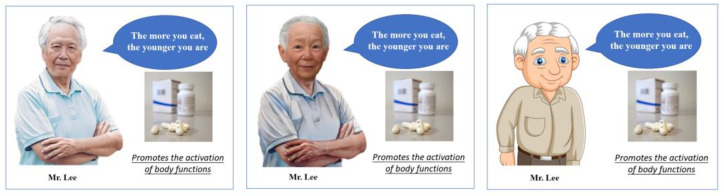
Endorser stimuli.

Study 1 was a single factor, between-subjects design (degree of realism of the endorser’s image: real human vs. realistic digital human vs. cartoonish digital human). A total of 205 students (55.6% female, M_age_ = 23.3) took part in the experiment. They were asked to imagine buying a health product — “Young Treasure”— for an elder in their family. The participants were first shown an advertisement endorsing a product for the elderly. As shown in Figure 2, the three groups of images were identical in all respects except for the category of the endorser.

Next, participants filled out questionnaires about the endorser’s perceived age and source credibility, as well as purchase intentions of the product. We use the Endorser-Credibility scale proposed by Ohanian [121], which includes attractiveness (5 items), trustworthiness (5 items), and expertise (5 items), with the sample items “This endorser appears to be good-looking”, “This endorser appears to be reliable”, and “This endorser appears to be an expert”. A 7-point Likert scale was used for the measurement (1 = strongly disagree, 7 = strongly agree). The alpha reliability of this scale was 0.928. The purchase intention question was adapted from Dodds’s [122]. The survey ended with the collection of demographic information, including gender and age.

### 4.2. Results

Manipulation check. A one-way ANOVA was performed to check the manipulation of the realism of the endorser’s image. There were significant differences in the participants’ perceived realism of the three types of endorsers (M_realhuman_ = 5.59, SD = 1.31; M_realistic_ = 3.28, SD = 1.91; M_cartoonish_ = 2.65, SD = 1.37; F (2202) = 67.889, *p* < 0.01), revealing a successful manipulation. The ANOVA also confirmed that the absence of any significant differences in attractiveness (M_realhuman_ = 4.01, SD = 1.12; M_realistic_ = 3.65, SD = 1.20; M_cartoonish_ = 3.89, SD = 1.07; F (2202) = 1,74, *p* > 0.05), trustworthiness (M_real_ = 3.72, SD = 1.46; M_realistic_ = 3.61, SD = 1.36; M_cartoonish_ = 3.80, SD = 1.42; *F*(2202) = 0.323, *p* > 0.05), and expertise (M_realhuman_ = 3.54, SD = 1.35; M_realistic_ = 3.45, SD = 1.27; M_cartoonish_ = 3.28, SD = 1.12; F (2202) = 0.76, *p* > 0.05) between the three types of endorsers.

Perceived age. The ANOVA revealed that participants’ perceived age of digital endorsers was significantly younger than that of real human endorsers (M_realhuman_ = 73.38, SD = 6.57; M_realistic_ = 71.49, SD = 6.61; M_cartoonish_ = 70.01, SD = 7.33; F (2202) = 4.399, *p* < 0.05). However, post hoc tests showed that there was no significant difference between the perceived age of cartoonish and realistic digital endorsers (*p* = 0.3). The implications of these results are twofold: firstly, they confirmed that digital endorsers can lead to a younger perceived age than real human endorsers in products for the elderly, in line with current trends in commercial practice; secondly, they excluded that perceived age plays a role in the different effects of the two types of digital endorsers’ advertisements.

**Figure 3:**
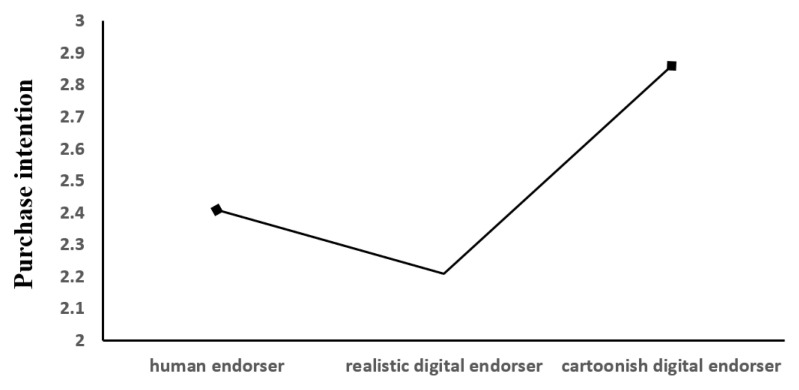
The main effect of endorser type on purchase intention.

Purchase intention. The ANOVA suggested that participants differed significantly in their willingness to purchase products with different types of endorsers (M_realhuman_ = 2.41, SD = 1.28; M_realistic_ = 2.21, SD = 1.20; M_cartoonish_ = 2.86, SD = 1.32; F (2202) = 4.67, *p* < 0.05; as depicted in Figure 3). Based on the post hoc test results, this was demonstrated by the fact that participants were significantly more likely to purchase a product endorsed by a cartoonish digital endorser than a real human endorser (*p* = 0.04 < 0.05) and a realistic digital endorser (*p* = 0.003 < 0.05). The results of this data validated H1.

Study 1 supported our hypothesis that cartoonish digital endorsers lead to higher purchase intentions (H1), even though cartoonish digital endorsers and realistic digital endorsers do not differ significantly in terms of perceived age and source credibility.

## 5. Study 2

Study 2 aimed to investigate the psychological mechanisms that influence participants’ purchase intentions when faced with products for older adults using different types of digital endorsers. We predicted that cartoonish digital endorsers with a low degree of image realism would lead to higher perceived social value, which in turn would increase purchase intention (H2). 

### 5.1. Participants and Procedures

We surveyed 200 participants on BBS in exchange for CNY 4 compensation. These participants included undergraduate students, postgraduate students, and teachers. We obtained 175 valid samples (53.7% females; M_age_ = 23.4) after removing 25 questionnaires that did not pass the attention test. The setting of endorsers in study 2 remained the same as in study 1, and the measures of image realism, credibility, and purchase intention were all the same as in study 1, with the addition of a measure of perceived value. We used a multidimensional perceived value scale [85] to measure consumers’ perceived functional value (5 items), perceived emotional value (5 items), and perceived social value (5 items) of a particular endorser’s product. Example items are “The health effects of this product are satisfactory”, “I am happy that my family can use this product”, and “I can earn the praise of others by buying this product “. A 7-point Likert scale was used for the measurement (1 = strongly disagree, 7 = strongly agree). The alpha reliability of this scale was 0.953.

### 5.2. Results

There were significant differences in the participants’ perceived realism of the three types of endorsers (M_realhuman_ = 5.60, SD = 1.38; M_realistic_ = 3.10, SD = 1.92; M_cartoonish_ = 2.58, SD = 1.44; F (2,172) = 60.72, *p* < 0.01), revealing a successful manipulation. Participants’ willingness to purchase products with different types of endorsers was significantly different (M_realhuman_ = 2.41, SD = 1.35; M_realistic_ = 2.16, SD = 1.17; M_cartoonish_ = 2.86, SD = 1.38; F (2172) = 4.43, *p* < 0.05), consistent with the results of Experiment 1. There was no difference in credibility between the different types of endorsers (*p* > 0.05).

**Figure 4:**
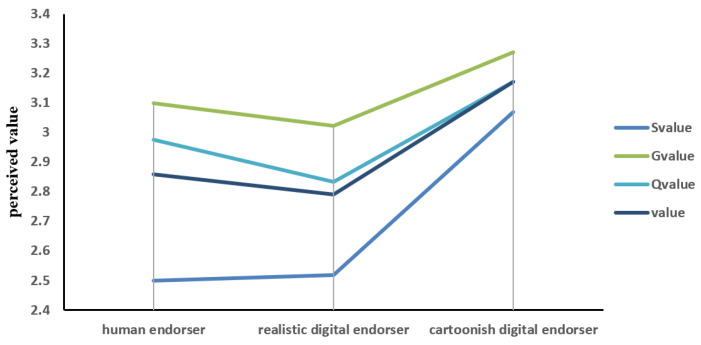
The relationship between digital endorser types and perceived value.

Then, ANOVA tests were conducted on perceived value, with endorser type as a factor. From the results (Figure 4), we know that there is no significant difference in overall perceived value, perceived functional value, and perceived emotional value among different types of endorsers (*p* > 0.05), but cartoon endorsers bring significantly higher perceived social value to participants (M_realhuman_ = 2.50, SD = 1.29; M_realistic_ = 2.51, SD = 1.34; M_cartoonish_ = 3.07, SD = 1.39; F (2172) = 3.38, *p* < 0.05).

**Figure 5:**
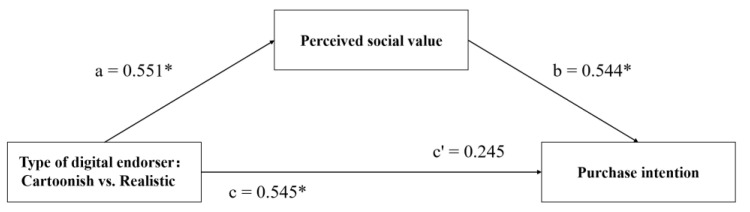
Path diagram. Note: * *p* < 0.05.

In the previous studies, real human endorsers as a control group did not differ significantly from realistic digital endorsers in terms of credibility, perceived social value, and purchase intention; therefore, in the follow-up study, only the differences between the two types of digital endorsers were considered. We recorded the digital endorser types, with cartoonish digital endorsers coded as 1 and realistic digital endorsers coded as 0. Next, we conducted the mediation analysis by model 4 in PROCESS within SPSS with 10,000 bootstrap samples and 95% bias-corrected intervals [123]. As shown in Table 1, perceived social value mediates the effect of digital endorser type on product purchase intention (*β* = 0.29; SE = 0.14; 95%CI [0.03, 0.59], not including 0), supporting H2 and H3. The result of the path analysis is depicted in Figure 5.

Study 2 replicates the results from Study 1 (H1) and also provides further evidence that cartoonish digital endorsers lead to a higher perceived social value compared to realistic digital endorsers, which in turn increases purchase intention (H2).

## 6. Study 3

Study 3 aims to find a simple and feasible way to mitigate the negative effects of increased image realism and enhance the preference for realistic digital endorsers.

Ad framing effects have been frequently used to change consumer perceptions and attitudes [21]; we predicted that the effectiveness of realistic digital endorsers would be elevated under prevention-focused (vs. promotion-focused) information (H3).

### 6.1. Participants and Procedures

A total of 127 participants (59.8% female, M_age_ = 23.2) recruited from BBS participated in a 2 (the type of digital endorser: realistic vs. cartoonish) × 2 (Ad information framing: promotion vs. prevention) between-subjects experiment. Participants were randomly distributed into four experimental groups (N_realistic, promotion_ = 32, N_realistic, prevention_ = 31, N_cartoonish, promotion_ = 32, N_cartoonish, prevention_ = 32). Firstly, they were instructed to view a picture of an advertisement for a product for the elderly. The promotional product continues the “Young Treasure” from study 1 and study 2, with the product description “Promotes the activation of body functions” and the tagline “The more you eat, the younger you are”. The name of the preventive product was changed to “Longevity Treasure”, the product description was changed to “Prevents degeneration of body functions”, and the slogan was changed to “You won’t age if you take it”. Next, participants completed the degree of image realism, perceived value (α = 0.954; M = 2.98, SD = 0.14), and purchase intention scales.

### 6.2. Results

Manipulation check. There were significant differences in the participants’ perceived realism of the two types of endorsers (M_realhuman_ = 5.60, SD = 1.38; M_realistic_ = 3.10, SD = 1.92; M_cartoonish_ = 2.58, SD = 1.44; F (2172) = 60.72, *p* < 0.01), revealing a successful manipulation. There was no difference in credibility between the different types of digital endorsers (*p* > 0.05).

Moderated mediation. We conducted a moderated mediation analysis by model 8 in PROCESS with 10,000 bootstrap samples and 95% bias-corrected intervals to test the full model. The independent variable was the type of digital endorsement, the dependent variable was purchase intention, the moderating variable was information framing, and the control variables were participants’ age and gender. As reported in Table 2, participants’ estimation of the social value of the digital endorser declines noticeably as its image grows more realistic (β = −1.277, *p* < 0.05). The relationship between digital endorser type **×** information framing type and perceived social value was not statistically significant (β = −1.326, *p* > 0.1). Once perceived social value is included in the model, as the digital endorser’s image becomes more realistic, purchase intentions may drop significantly (β = −1.661, *p* < 0.05). Purchase intention is significantly predicted by perceived social value (β = 0.466, *p* < 0.001). Meanwhile, the relationship between digital endorser type **×** information framing type and purchase intention was statistically significant (β = 0.982, *p* < 0.05). The data results indicate that information framing and digital endorser type have an interactive effect on purchase intention.

**Figure 6:**
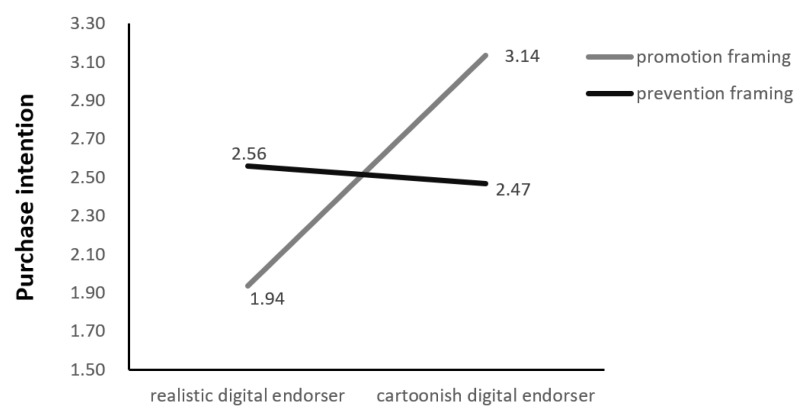
Moderating effect of information framing.

We further conducted a two-way ANOVA on purchase intention, with the type of digital endorser (realistic vs. cartoonish) and information framing (promotion vs. prevention) as fixed factors, and illustrated the results in Figure 6. Consistent with the results of the moderated mediation analysis, the digital endorser type × advertising framing type interaction was significant (F (1126) = 19.172, *p* < 0.05, η2 = 0.08). Information framing moderates the effect of endorser type (realistic vs. cartoonish) on purchase intention. For elderly products in the promotional information framing, the cartoonish endorser brings higher purchase intention; however, in the preventive information framing, realistic digital endorsers have a more positive impact on purchase intention, and the two endorsers’ performances are no longer noticeably different.

Study 3 further replicated the mediating effect of perceived social value between the type of digital endorser and purchase intention. We also demonstrated that the negative effects of image realism on digital endorsers could be mitigated by manipulating the advertising information framing with a few simple changes in textual descriptions.

## 7. Discussion

### 7.1. Theoretical Implications

This research has made several important theoretical contributions. Firstly, our study enriches the literature on advertising for elderly products. Advertising research on products for the elderly has been a neglected subject [8]. There are also controversial arguments regarding the function of older endorsers in advertising [39]. On the one hand, our research offers experimental support for the commercial practice of an endorser with a young appearance performing better in older product advertising. On the other hand, we examine the high-frequency buying scenario of elderly products, i.e., the gift-giving scenario, and propose that an endorser with a non-controversial image and a higher perceived social value is a factor that increases purchase intention. Previous research on endorsers has primarily relied on source-credibility models [23,25] and match-up models [3]. This study enriches the literature from the subjective perspective of the purchaser by suggesting that endorsers need to allow purchasers to maximize the meaning of their purchases without ambiguity.

Secondly, this study expands the current literature on digital humans in advertising research. “Digital endorsers” is a developing area of academic research. Most previous research was primarily qualitative, based on the case and content analysis, and focused on the source credibility and interaction style established by digital people on social media [16], contrasting the advantages and disadvantages of digital endorsers to real human endorsers [124,125]. Our study is one of the first empirical studies to incorporate digital humans into traditional product advertising. This study not only provides a theoretical foundation for the greater use of digital humans in business, but it also explains the processes of the function of a digital human in the advertising of elderly products. Furthermore, we respond to previous contradictory views on the degree of realism of the digital endorser’s image [53,126]. When consumers are more interested in the product’s perceived social value, less realistic digital endorsers can generate higher effectiveness.

Finally, we explored the interaction effect between the construal level of the endorser’s image and information framing. We discovered that preventive information framing allows consumers to analyze the strengths and flaws of endorsers more comprehensively in the process of facial recognition of advertising endorsers, rather than merely relying on thin-slice assessments [74], boosting consumers’ risk tolerance [105].

### 7.2. Practical Implications

This study offers marketing insights into the growing markets for digital human endorsements and advertising of elderly products. On the one hand, when selecting endorsers for elderly products, particularly those commonly found in gifting scenarios, it is critical not to prioritize personalization or novelty but rather to select endorsers on whom people can easily create a social consensus, allowing buyers to more securely and accurately express the meaning of the gift to their elders (the recipients of the gift) without misunderstanding or ambiguity. On the other hand, from the perspective of digital human design, we propose that diminishing the realism of the digital endorser image can make digital endorsers work better in the advertising of elderly products, such that there is no need to strive for a close match to actual humans when building digital endorsers.

Additionally, advertisers can adjust the message framing of their advertising to match different categories of digital endorsers. While cartoonish digital endorsers perform better in general, realistic digital endorsers can have a wide range of uses in more scenarios [97,127]. Therefore, if businesses want to enhance the use of realistic digital endorsers, product advertising text can be developed with a preventive information framing, such as more risk-related words, to grab customers’ attention and effectively highlight the value of realistic digital endorsers.

### 7.3. Limitations and Future Research

This research has several limitations that represent avenues for future research. Firstly, the majority of our participants were students, and younger participants may be more familiar and comfortable with cartoonish digital endorsers. Although we excluded the effect of multidimensional credibility and found no differences in the perceived emotional value dimension, there may still be some hidden influences. Future research should cover all age groups in the experimental sample to improve the robustness of the results. Secondly, in the current experimental materials, the perceived age of the elderly endorsers, whether real or digital, is around 70 years old. However, the classification of the elderly is becoming more nuanced, with young elderly and super elderly creating different perceptions among consumers. Therefore, in future research, we will change the experimental stimulus materials to explore the influence of endorsers’ perceived age on purchase intention. In addition, we have only focused on health products, which are typical elderly products. Products for the elderly are available in a much wider variety today, including technological ones (e.g., smart watches that monitor the wearer’s health) and cultural ones (e.g., educational courses for the elderly). The issue of matching various product categories and digital endorsers needs to be further studied.

## 8. Conclusions

In conclusion, this research explores the use of digital endorsers in advertising for elderly products, as well as the mediation mechanisms underlying the influence, to help companies in selecting more appropriate digital endorsers to drive more transactions. Compared with realistic digital endorsers, we discovered that cartoonish digital endorser images had a higher construal level and led to higher purchase intentions. This effect is driven by perceived social value. When buying products for the elderly, one of the most important considerations for buyers is perceived social value [88]. Social value stems from social identity or the cultivation of a social self-concept [85]. When the image of the endorser is too realistic, it is difficult to have consistent attitudes and opinions between groups [19]. The difference in the impact of the two types of digital endorsers in advertising can be eliminated by modifying the information framing of the ad. when product advertisements are presented in a promotional framing, consumers will rely more on intuitive judgments and ignore objective conditions [106], thus producing more positive results for digital endorsers with a low degree of image realism. In the preventive framing context, consumers attentively and thoroughly examine digital endorsers, thereby decreasing the differences in purchase intentions caused by the level of image realism.

## Figures and Tables

**Table 1:** Regression analysis of the mediating effect.

Effect (X → Svalue → PI)	B	SE	LLCI	ULCI
Direct effect of X on Y	0.25	0.19	−0.14	0.63
Indirect effect of X on Y	0.29 *	0.14	0.03	0.59
Total effect of X on Y	0.54 *	0.23	0.08	1.01

* *p* < 0.05

**Table 2:** Results of moderated mediation analysis.

	Perceived Social Value		Purchase Intention	
	β	t	β	t
X (cartoonish vs. realistic)	−1.277 *	−1.681	−1.661 *	−3.124
Perceived social value			0.466 **	7.390
information framing (promotion vs. prevention)	−1.326	−1.082	−2.302 *	−2.957
X×information framing	0.456	0.953	0.982 *	2.957
Age	−0.021	0.045	0.003	0.112
Gender	−0.487 *	−1.945	−0.540 *	−3.082
R^2^	0.108		0.684	
F	2.892 *		17.453 **	

Note: * *p* < 0.05, ** *p* < 0.01.

## Data Availability

Data supporting reported results are available from the authors on request.
